# Removals and Appointments

**Published:** 1856-11

**Authors:** 


*Removals and Appointments.*—During the past season, the Buffalo Med[illegible]al Association has lost a number of members by removal. Among them [illegible] our friend Dr. J. C. Lay, who goes to Dubuque, Iowa, bearing with him th[illegible] good wishes of all his professional associates here.

Dr. Holmes, also a young gentleman of talent, has removed to the west, leaving his place as Clinical Assistant to the Professor of Clinical Medicine and Pathology in the University of Buffalo, vacant.

Dr. Austin M. Nichols has been appointed to the vacant post. Dr. Nichols is a man of industry and talent, and will fulfill his duties in the most competent manner.

